# A systematic review of the effectiveness of individual, community and societal level interventions at reducing socioeconomic inequalities in obesity amongst children

**DOI:** 10.1186/1471-2458-14-834

**Published:** 2014-08-11

**Authors:** Frances C Hillier-Brown, Clare L Bambra, Joanne-Marie Cairns, Adetayo Kasim, Helen J Moore, Carolyn D Summerbell

**Affiliations:** Department of Geography, Wolfson Research Institute for Health and Wellbeing, Durham University Queen’s Campus, Stockton-on-Tees, TS17 6BH UK; School of Medicine, Pharmacy and Health, Wolfson Research Institute for Health and Wellbeing, Durham University Queen’s Campus, Stockton-on-Tees, TS17 6BH UK; Wolfson Research Institute for Health and Wellbeing, Durham University Queen’s Campus, Stockton-on-Tees, TS17 6BH UK

**Keywords:** Obesity, Socioeconomic status, Inequalities, Infant, Child, Adolescent, Interventions

## Abstract

**Background:**

Tackling childhood obesity is one of the major contemporary public health policy challenges and vital in terms of addressing socioeconomic health inequalities.

We aimed to systematically review studies of the effectiveness of interventions (individual, community and societal) operating via different approaches (targeted or universal) in reducing socio-economic inequalities in obesity-related outcomes amongst children.

**Methods:**

Nine electronic databases were searched from start date to October 2012 along with website and grey literature searches. The review examined the best available international evidence from interventions that aimed to prevent obesity, treat obesity, or improve obesity-related behaviours (diet and/or physical activity) amongst children (aged 0-18 years) in any setting and country, so long as they provided relevant information and analysis on both socioeconomic status and obesity-related outcomes. Data extraction and quality appraisal were conducted using established mechanisms and narrative synthesis was conducted.

**Results:**

We located 23 studies that provided the ‘best available’ (strongest methodologically) international evidence. At the individual level (n = 4), there was indicative evidence that *screen time reduction* and *mentoring health promotion* interventions could be effective in reducing inequalities in obesity. For the community level interventions (n = 17), evidence was inconclusive - with some studies suggesting that *school-based health promotion activities* and *community-based group-based programmes* were effective in reducing obesity - others not. Societal level evaluations were few (n = 1). However, there was no evidence to suggest that any of these intervention types increase inequalities and several studies found that interventions could at least prevent the widening of inequalities in obesity. The majority of studies were from America and were of 6-12 year old children.

**Conclusions:**

The review has found only limited evidence although some individual and community based interventions *may* be effective in reducing socio-economic inequalities in obesity-related outcomes amongst children but further research is required, particularly of more complex, societal level interventions and amongst adolescents.

**Electronic supplementary material:**

The online version of this article (doi:10.1186/1471-2458-14-834) contains supplementary material, which is available to authorized users.

## Background

Persistently high levels of childhood overweight and obesity globally, and the associated health complications, have been well documented [1–3]. In high income countries, evidence from epidemiological studies have continually shown that obesity levels are higher in children of the lowest socioeconomic status [4–10]. Addressing inequalities in obesity therefore has a very high profile on the public health agenda internationally. There is also concern that interventions aiming to prevent and treat obesity are taken up more effectively by the most advantaged groups and therefore widen inequalities in obesity even further [8].

Some effective universal public health interventions may increase inequalities by disproportionately benefitting less disadvantaged groups (‘intervention-generated inequalities’ or IGIs) [11]. Such IGIs may arise at a number of points in the implementation of an intervention, including intervention efficacy, service provision or access, uptake, and compliance [12]. There is a need to understand which types of interventions are likely to produce IGIs, and which can reduce inequalities. There is a substantial body of theoretical work and guidance on the kinds of interventions which are likely to reduce or increase inequalities [13–15], and Lorenc [16] has conducted a rapid overview of systematic reviews to identify the types of interventions that are more likely to produce IGIs, and which have the potential to reduce inequalities. Lorenc [16] found that media campaigns and workplace smoking bans show some evidence of increasing inequalities (IGIs) between socioeconomic status groups. Data published on IGIs were lacking. However, structural workplace interventions, provision of resources and fiscal interventions such as tobacco pricing showed some evidence of reducing inequalities. Lorenc [16] concluded that their findings are consistent with the idea that ‘downstream’ preventative interventions are more likely to increase health inequalities than ‘upstream’ interventions. A subsequent systematic review of universal interventions to reduce smoking confirms these findings; price/tax increases had the most consistent positive equity impact [17].

One would expect that effective targeted (at those most disadvantaged) public health interventions, in contrast, avoid the problem of IGIs. Indeed, this has recently been confirmed by Barr [18] who investigated whether the policy of increasing National Health Service funding to a greater extent in deprived areas of England, compared with more affluent areas, led to a reduction in inequalities in mortality amenable to health care [18]. Using data from a longitudinal ecological study from 2001 to 2011, Barr found that the policy was associated with a reduction in absolute health inequalities. Similar evidence for obesity-related outcomes is scarce. Magnee [19] reviewed the equity-specific effects of 26 Dutch obesity-related lifestyle interventions (of variable quality and design) but findings were inconsistent. However, a recently published study from Alberta, Canada supports these findings; the quasi-experimental trial found that a ‘whole school-based’ physical activity promoting intervention targeted at those most disadvantaged, which took an ‘upstream’ approach, reduced inequalities in physical activity [20].

It is also possible that the way in which a complex intervention is organised and implemented (i.e. context) can impact on its ability to reduce inequalities [21]. For example, a recent systematic review by Durand [22] suggests that interventions which involve shared decision-making (and increase participant engagement) may be more beneficial to disadvantaged groups compared with those of higher literacy/socioeconomic status.

Existing systematic reviews only examine the effects of interventions that reduce *overall* levels of obesity, as opposed to the effects on *inequalities* in obesity. Therefore, there is a lack of accessible policy ready evidence on what works in terms of interventions to reduce inequalities in childhood obesity. Further, there is increasing recognition amongst policy makers that to effectively tackle complex health problems, such as obesity, and to reduce health inequalities requires integrated policy action across different intervention levels (individual, community, society), as well as across the life course (starting with childhood) [23, 24].

### How interventions can impact inequalities in childhood obesity

Interventions can be characterised by their *level* of action and their *approach* to tackling inequalities. Whitehead [25] describes four *levels* of interventions to tackle inequalities: strengthening individuals (person based strategies to improve the health of disadvantaged individuals), strengthening communities (improving the health of disadvantaged communities and local areas by building social cohesion and mutual support - via collective activities), improving living and school environments (reducing exposure to health-damaging material and psychosocial environments across the whole population), and promoting healthy macro policy (improving the macro-economic, cultural and environmental context that influence the standard of living achieved by the whole population). According to Graham and Kelly [14], these interventions are underpinned by one of three different *approaches* to health inequality: disadvantage (improving the absolute position of the most disadvantaged individuals and groups), gap (reducing the relative gap between the best and worst off groups), or gradient (reducing the entire social gradient). Interventions are thus either *targeted* (directed at those who are disadvantaged) or *universal* (interventions that influence the entire social gradient).

The aim of this review was to systematically examine the effectiveness of interventions (individual, community and societal) operating via different approaches (targeted or universal) in reducing socio-economic inequalities in obesity-related outcomes amongst children. A companion paper examines interventions for reducing socioeconomic inequalities in obesity amongst adults [26].

## Methods

Our review was carried out following established criteria for the good conduct and reporting of systematic reviews. The full review protocol is published elsewhere [27] and is registered with PROSPERO (CRD42011001740). The full review is available to view at http://www.phr.nihr.ac.uk/funded_projects/obesity.asp [28].

### Data sources

The following electronic databases were searched from their respective start dates up to the 11^th^ October 2012: MEDLINE, EMBASE, CINAHL, PsycINFO, Social Science Citation Index, ASSIA, IBSS, Sociological Abstracts and the NHS Economic Evaluation Database (see Additional file 1 for the full search strategies). We did not exclude papers on the basis of language, country or publication date. The electronic database searches were supplemented with website and grey literature searches.

### Types of intervention

Our review examined interventions at the individual, community and societal (environment and macro policy) level that might reduce inequalities in obesity-related outcomes amongst children aged 0-18 years. Interventions that aimed to prevent obesity, treat obesity, or improve obesity-related behaviours (diet and/or physical activity) were considered relevant, so long as they provided relevant information and analysis on both socioeconomic status and obesity-related outcomes. We defined individual level interventions as those that included individualised/one-to-one health promotion, education, advice, counselling or subsidy and were conducted in a health care or research setting, or in participant’s homes. Community level interventions were defined as group-based health promotion, education, advice, counselling or subsidy only interventions, or interventions conducted in a community setting (for example a school, community centre, sports centre and shop). *We have classified the group-based educational interventions as community, rather than individual interventions, using ‘the intent’ of the intervention as an aid to classification* [29] *. Although we acknowledge that an element of the intent of these types of interventions is to strengthen individuals (increase ‘agency’) by targeting behaviour change, we believe that the main intent is to target the condition (group setting, peer support, peer pressure, etc) in which behaviour occurs. We do acknowledge that it is a grey area for these types of studies.* Societal level studies were split into two sub-groups: Societal-environment level interventions as those that included a change in environment or access to environment; and Societal-policy level interventions as macro-level policies such as taxation, advertising restriction or subsidies. Interventions were also classified in terms of whether they took a gradient approach and included participants of all socio-economic status (SES) (“universal” interventions) or a targeted approach i.e. aimed at low SES participants only (“targeted” interventions). Measures and proxy measures of SES were parental income, parental education, parental occupation, area level or school level disadvantage (for example number of pupils receiving free or reduced school meals). We did not include ethnicity (or faith or culture) as a measure of SES. Interventions that involved drugs or surgery, and laboratory-based studies, were excluded from the review.

Our review considered prevention and treatment interventions that might reduce socioeconomic inequalities in the prevalence of obesity-related outcomes (i.e. effective interventions targeted at low SES children, or universal interventions that work equally or more effectively in low SES children compared with high SES children).

### Types of studies

Our full review [28] included randomised controlled trials (RCTs) and non-randomised controlled trials (classified as experimental studies) that included either a non-treatment control group or standard treatment group, and prospective and retrospective cohort studies, with or without control/standard treatment groups, and prospective repeat cross-sectional studies with or without control/standard treatment groups (classified as observational studies). Only studies with duration of at least 12 weeks (combination of intervention and follow up) were included. The justification for using a 12 week cut-off was a pragmatic one, in that most existing interventions and initiative, particularly those which are school based, are of a shorter duration, for example over one school term. Given that the aim of our review was to provide useful information for policy makers and commissioners of services, who will be mindful of costs (driven, in part, by duration of the intervention), we did not want to exclude such interventions from our review; the same criteria (and justification) was used in a Cochrane review on interventions to prevent obesity in children [30]. That said, we appreciate that longer term (e.g. one year) changes in obesity-related outcomes would provide a more confident assessment of the effectiveness of such interventions. For the purpose of this article only the best evidence available for each intervention level is reported and, therefore, only RCTs and non-randomised controlled trials (experimental studies) were included.

### Types of outcome measure

Studies were included if they reported an obesity-related outcome (e.g. weight and height; body mass index; waist measurement/waist to hip proportion; percentage body fat; skin fold thickness; ponderal index) and if they examined differential effects with regard to socio-economic status, or were targeted specifically at disadvantaged groups or were conducted in deprived areas.

### Data extraction and quality appraisal

The initial screening of titles and abstracts was conducted by one reviewer with a random 10% of the sample checked by a second reviewer. Data extraction was conducted by one reviewer using established data extraction forms and independently checked by a second reviewer. The methodological quality of the included studies was appraised independently by two reviewers using the Cochrane Public Health Review Group recommended Effective Public Health Practice Project Quality Assessment Tool for Quantitative Studies [31]. This tool includes, amongst other things, an examination of sampling strategy, response and follow-up rates, intervention integrity, statistical analyses and assessment of adjustment for confounders. We used the quality appraisal criteria for descriptive purposes and to highlight variations between studies. Any discrepancies were resolved through discussion between the authors and, if consensus was not reached, with the project lead.

### Analysis and synthesis

Our full review [28] used broad study inclusion criteria and conducted a wide search in order to capture the entire evidence base on the effects of interventions to reduce inequalities in obesity-related outcomes amongst children. This resulted in a very large evidence base that was much larger than anticipated. To make sense of it for policy and practice, this article focuses only on a narrative synthesis of the ‘best available’ international evidence for each intervention type. Best available evidence was defined in terms of both study design and study quality by each intervention type so that only those studies that provided the highest quality for each intervention type are synthesised in this paper.

## Results

**Figure 1:**
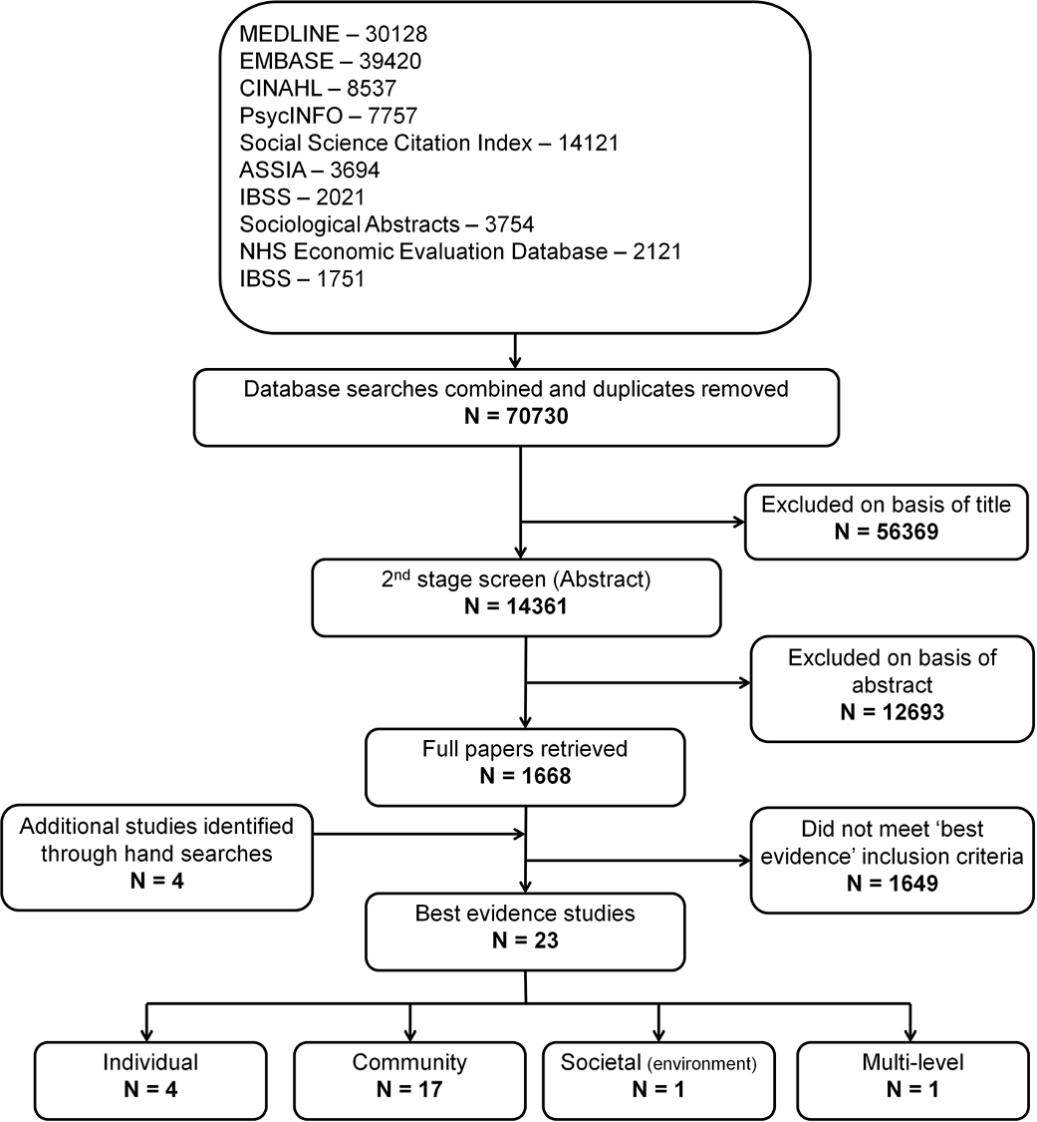
**QUOROM statement flow diagram.**

Our database searches identified 70730 records (Figure 1). After title and abstract screening 1668 papers were retrieved. Supplementary searching revealed an additional four studies that met the inclusion criteria for this review. After full paper screening, the ‘best available’ evidence for each intervention level was obtained from 23 studies (4 individual level, 17 community, 1 societal-environmental and 1 multi-level interventions).

For the individual level interventions, the ‘best available’ evidence is provided by moderate quality, experimental studies (randomised and non-randomised controlled trials, randomised and non-randomised cluster trials). For the community level and multi-level interventions, this was provided by strong quality experimental studies. Finally, moderate quality experimental studies were the strongest identified for the societal-environmental level interventions. The descriptions and findings of the ‘best available’ evidence studies are summarised in Tables 1, 2, 3, 4. Please see (Additional file 2: Tables S1-S4 for effect sizes - where data are available).

**Table 1:** **Summary details of individual level studies included in the review**

***Study***	***Design & quality Appraisal*** ^***1***^	***Setting & participants***	***Study aim***	***Intervention*** ^***2***^	***Inequality*** ^***3***^	***Summary results*** ^***4***^ ***↑ = increase ↓ = decrease ↔ = no change***		***Impact on inequalities in obesity*** ^***5***^
***Individual level interventions***								
Taveras et al 2011 [32]	Cluster RCT; 1 year follow-up; Final sample = 445; Quality = Moderate	10 primary care paediatric centres, USA; 2-6 years; 48% girls; Obese and high risk of obese	Reduction of BMI in obese and risk of obese children	Nutrition and physical activity intervention; Treatment: Weight management programme (High Five for Kids) – diet and physical activity education and counselling, and behavioural cognitive therapy	**Universal:** results analysed by household income	BMI (low income)	↓	+
						BMI (high income)	↔	
Wake et al 2009 [33]	RCT; 12 month follow-up; Final sample = 245; Quality = Moderate	45 family medical practices, Australia; 5-10 years; 61% girls; Overweight or mildly obese	Reduce BMI gain in overweight or mildly obese children	Nutrition and physical activity intervention; Treatment: Primary care obesity management programme (LEAP2) – screening for overweight/obesity followed by GP administered counselling (diet and physical activity)	**Universal:** SES did not modify any intervention effect	BMI	↔	0
						Waist circumference	↔	
						Prevalence overweight/ obese	↔	
Epstein et al 2008 [34]	RCT; 24 month follow-up; Final sample = 67; Quality = Moderate	Participant’s homes, USA; 4-7 years; ≥75^th^ percentile (at risk of overweight/ overweight/obese)	Reduction of obesity-related sedentary behaviours in children at risk of obesity	Physical activity intervention; Treatment/Prevention: Intervention to reduce TV viewing and computer use – duration of use regulated; monetary incentives for reduced use; and newsletters containing information and advice	**Universal:** intervention effect compared between low SES and high SES groups	BMI z score (low SES)	↓	+
						BMI z score (high SES)	↔	
Black et al 2010 [35]	RCT; 24 month follow-up; Final sample = 179; Quality = Moderate	Homes and community sites (e.g. parks and convenience stores), USA; 11-16 years; 49% girls	Health promotion and prevention of obesity	Nutrition and physical activity intervention; Prevention: Mentor-based health promotion and obesity prevention programme (Challenge!) – Session with mentors including food preparation, exercise; goal setting, progress discussions, and provision of information and recipes. Rap music video promoting healthy eating and physical activity	**Targeted:** low-income communities	Prevalence overweight/ obese	↓	+
						BMI z score	↔	
						Ideal weight:		
						% body fat	↔	
						Fat mass	↔	
						Fat-free mass	↔	
						Obese/overweight:		
						% body fat	↓	
						Fat mass	↓	
						Fat-free mass	↑	

^1^Global Quality appraisal from EPHPP (16); ^2^Prevention or treatment intervention; ^3^Targeted/Universal approach to inequality; ^4^p < 0.05.This is the relative mean differences between intervention and control at follow-up; ^5^+ positive intervention effect so it reduces obesity-related outcomes in low SES groups or reduces the SES gradient in obesity-related outcomes, 0 no intervention effect or no effect on SES gradient in obesity-related outcomes; *SES* = Socioeconomic status; *BMI* = Body mass index.

**Table 2:** **Summary details of community level studies included in the review**

***Study***	***Design & quality appraisal*** ^***1***^	***Setting & participants***	***Study aim***	***Intervention*** ^***2***^	***Inequality*** ^***3***^	***Summary results*** ^***4***^ ***↑ = increase ↓ = decrease ↔ = no change***		***Impact on inequalities in obesity*** ^***5***^
Kain et al 2004 [36]	Non-randomised cluster controlled trial; 6 month follow-up; Final sample = 3086; Quality = Strong	5 Schools, Chile; 10.6 years; 47% girls	Reduction and prevention of obesity in low SES children	Nutrition and physical activity intervention; Prevention: nutrition education (children and parents), extra time in PE lessons, encouragement of PA during daily recess, healthy snacks in vending machines (voluntary), incentives for healthy eating and sports equipment for schools	**Targeted:** Low SES schools (35% children receiving School Lunch Program)	BMI z score (boys)	↓	+ (boys)
						Triceps skinfold (boys)	↔	
						Waist circumference (boys)	↔	
						BMI z score (girls)	↔	
						Triceps skinfold (girls)	↔	
						Waist circumference (girls)	↔	
Jansen et al 2011 [37]	Cluster RCT; 8 month follow-up; Final sample = 2416; Quality = Strong	20 Schools, The Netherlands; 6-12 years; 51% girls	Weight reduction and prevention of obesity in low SES children	Nutrition and physical activity intervention; Prevention: nutrition, activity living and healthy lifestyle education, 3 PE lessons per week and voluntary additional after-school sport and play activities	**Targeted:** Low income inner-city, multi-ethnic schools	Children 6-9 years:		+ (6-9 years)
						BMI	↔	
						Waist circumference	↓	
						Prevalence overweight	↓	
						Children 9-12 years:		
						BMI	↔	
						Waist circumference	↔	
						Prevalence overweight	↔	
Nemet et al 2011 [38]	Cluster RCT; 1 year follow-up; Final sample = 297; Quality = Strong	11 Kindergartens, Israel; 4.2-6.5 years; 45% girls	Prevention of obesity	Nutrition and physical activity intervention; Prevention: Nutrition education classes and flyers for parents; exercise sessions and songs related to topic of nutrition and exercise	**Targeted:** kindergarten in low SES communities	BMI (boys)	↓	+ (boys)
						BMI% (boys	↓	
						BMI (girls)	↔	
						BMI% (girls)	↔	
Bingham 2002 [39]	Cluster RCT; 1 year follow-up; Final sample = 985; Quality = Strong	12 schools, USA; 8-10 years; 51% girls	CVD risk factor reduction	Nutrition and physical activity intervention; Prevention: CVD risk factor reduction intervention – education (including nutrition and physical activity) and physical activity sessions	**Universal:** SES was not found to be a moderator of the intervention effect	Skinfold thickness	↓	0
Simon et al 2008 [40]	Randomised cluster trial; 48 month follow-up; Final sample = 732; Quality = Strong	8 schools, Eastern France; 11-12 years; 50% girls	Increase physical activity by changing attitudes, promoting the social support of parents and teachers, making the environment more supportive of physical activities	Physical activity intervention; Prevention: physical activity education and increased physical activity classes, ‘cycling to school’ days and sports events	**Universal:** no differences in results by parental occupation	BMI	↓	0
						Physical activity	↑	
						TV/video use	↓	
Bellows 2007 [41]	Cluster RCT; 18 week follow-up; Final sample = 201; Quality = Strong	4 Head Start centres, USA; 3-5 years; 46% girls	Prevent obesity	Nutrition and physical activity intervention; Prevention: Food Friends Get Movin’ with Mighty Moves^TM^ intervention – physical activity sessions and nutrition education	**Targeted:** low-income, ethnic minority preschoolers	BMI z score	↔	0
de Meij et al 2011 [42]	Cluster non-randomised control trial; 20 month follow-up; Final sample = 2064; Quality = Strong	19 schools, The Netherlands; 6-12 years; 50% girls	To increase physical activity among children living in socially and economically deprived areas	Physical activity intervention; Prevention: physical activity education and exercise sessions	**Targeted:** majority of pupils low SES	BMI	↔	0
						Waist circumference	↔	
						Organised sport participation	↑	
						Physical activity	↔	
						Fitness	↔	
Herrick et al 2012 [43]	Cluster non-randomised controlled trial; 5 month follow-up; Final sample = 98; Quality = Strong	6 schools, USA; 10-11 years; 55% girls	Increase physical activity levels	Physical activity intervention; Prevention: after-school physical education sessions; self-management education	**Targeted:** largely low-income population	BMI	↔	0
						BMI z score	↔	
						MVPA	↔	
Lubans et al 2012 [44]	Cluster RCT; 12 month follow-up; Final sample = 294; Quality = Strong	12 schools, Australia; 13.2 years; 100% girls	Prevention of unhealthy weight gain in low SES adolescent girls	Nutrition and physical activity intervention; Prevention: Nutrition and Enjoyable Activity for Teen Girls (NEAT Girls) – nutrition education; exercise sessions; self-monitoring; social support	**Targeted:** schools in low-income communities	BMI	↔	0
						BMI z score	↔	
						Body fat%	↔	
Sichieri et al 2008 [45]	Cluster RCT; 8 month follow-up; Final sample = 927; Quality = Strong	22 schools, Brazil; 10-11 years; 53% girls	Prevention of excess weight gain	Nutrition intervention; Prevention: educational intervention to reduce consumption of sugar-sweetened beverages and encourage water consumption	**Targeted:** children from low SES families	BMI (overall)	↔	+ (overweight girls)
						BMI (overweight girls)	↓	
Walter et al 1985 [46]	Cluster RCT; 1 year follow-up; Final sample = 1115; Quality = Strong	22 Schools, USA; 9 years; 49% girls	Prevention of chronic disease risk factors (including obesity)	Nutrition and physical activity intervention; Prevention: “Know Your Body” curriculum focusing on nutrition physical fitness and smoking prevention	**Targeted:** Children from low income families	Ponderosity index	↔	0
						Triceps skinfold thickness	↔	
Robinson 1999 [47]	Randomised cluster trial; 6 month follow-up; Final sample = 192; Quality = Strong	2 schools, USA; 8-9 years; 45% girls	Prevent the onset of obesity	Physical activity intervention; Prevention: education course to reduce TV and video game use including a 10 day TV turn off. Home TV usage monitor. Parental education materials	**Universal:** no differences in results by parental education	BMI	↓	0
						Triceps skin fold thickness	↓	
						Waist circumference	↓	
						Waist-hip ratio	↓	
Kalavainen et al 2007 [48]	RCT; 12 month follow-up; Final sample = 69; Quality = Strong	1 Health care centre, Finland; 7-9 years; 60% girls; Obese	Treatment of obesity	Nutrition and physical activity intervention; Treatment: Family-based group treatment programme – diet and physical activity education and behavioural therapy	**Universal:** No association between social class and obesity-related outcomes	Weight for height	↓	0
						BMI	↓	
						BMI SDS	↔	
Alves et al 2008 [49]	RCT; 6 month follow-up; Final sample = 68; Quality = Strong	Community setting (exact setting unclear), Brazil; 5-10 years; 49% girls; Overweight	Increase physical activity in overweight children to reduce BMI	Physical activity intervention; Treatment: Physical activity sessions 3 times per week	**Targeted:** Children from a disadvantaged area	BMI	↓	+
Robinson et al 2003 [50]	RCT (pilot); 12 week follow-up; Final sample = 60; Quality = Strong	Community centres and homes, USA; 8-10 years; 100% girls; At risk of obesity	Prevent further weight gain in low SES African American girls	Physical activity intervention; Treatment: Dance classes and TV viewing reduction intervention (GEMS) targeting African American girls at risk of obesity	**Targeted**: Recruited from low income neighbourhoods	BMI	↔	0
						Waist circumference	↔	
Willet 1995 [51]	Non-randomised controlled trial; 1 year follow-up; Final sample = 40; Quality = Strong	1 community setting (exact setting unclear), USA; 7-12 years; 100% girls	Prevention of obesity in low income African American girls	Nutrition and physical activity intervention; Prevention: Mother and daughter culturally specific obesity prevention programme (based on the Know Your Body health education curriculum)	**Targeted:** low SES, African American girls	BMI	↔	0
						% overweight	↔	
Hamad et al 2011 [52]	RCT; 1 year follow-up; Final sample = 1501; Quality = Strong	Microcredit institution, Republic of Peru; <5 years	To improve the general health of disadvantaged children	Nutrition and physical activity intervention; Prevention: Microcredit loan with the addition of health education sessions to parents	**Targeted:** children of families receiving microcredit	BMI	↔	0
						% overweight	↔	

^1^Global Quality appraisal from EPHPP (16); ^2^Prevention or treatment intervention; ^3^Targeted/Universal approach to inequality; ^4^p < 0.05.This is the relative mean differences between intervention and control at follow-up; ^5^+ positive intervention effect so it reduces obesity-related outcomes in low SES groups or reduces the SES gradient in obesity-related outcomes, 0 no intervention effect or no effect on SES gradient in obesity-related outcomes; *SES* = Socioeconomic status; *BMI* = Body mass index; *MVPA* = Moderate to vigorous intensity physical activity.

**Table 3:** **Summary details of the societal level study included in the review**

***Study***	***Design & quality appraisal*** ^***1***^	***Setting & participants***	***Study aim***	***Intervention*** ^***2***^	***Inequality*** ^***3***^	***Summary results*** ^***4***^ ***↑ = increase ↓ = decrease ↔ = no change***		***Impact on inequalities in obesity*** ^***5***^
Bürgi et al 2012 [53] and Puder et al 2011 [54]	Cluster RCT; 9.5 month follow-up; Final sample = 625; Quality = Strong	40 schools, Switzerland; 5.2 years; 50% girls	Reduce obesity and improve fitness levels in children from socially disadvantaged backgrounds	Nutrition and physical activity intervention; Prevention: Built environment adapted to promote physical activity (fixed and mobile equipment) plus exercise sessions; nutrition education; information and discussion evenings for parents	**Universal:** trend for greater intervention effectiveness in higher SES children but not statistically significant	BMI	↔	0
						Body fat% (↑SES)	↓	
						Body fat (↓SES)	↔	
						Skinfold thickness	↓	
						Waist circumference	↓	
						Overweight prevalence	↔	
						Fitness (↑SES)	↑	
						Fitness (↓SES)	↔	

^1^Global Quality appraisal from EPHPP (16); ^2^Prevention or treatment intervention; ^3^Targeted/Universal approach to inequality; ^4^p < 0.05.This is the relative mean differences between intervention and control at follow-up; ^5^+ positive intervention effect so it reduces obesity-related outcomes in low SES groups or reduces the SES gradient in obesity-related outcomes, 0 no intervention effect or no effect on SES gradient in obesity-related outcomes; *SES* = Socioeconomic status; *BMI* = Body mass index.

**Table 4:** **Summary details of the multi-level study included in the review**

***Study***	***Design & quality appraisal*** ^***1***^	***Setting & participants***	***Study aim***	***Intervention*** ^***2***^	***Inequality*** ^***3***^	***Summary results*** ^***4***^ ***↑ = increase ↓ = decrease ↔ = no change***		***Impact on inequalities in obesity*** ^***5***^
Sanigorski et al 2008 [55]	Quasi-experimental including cluster RCT; 3 year follow-up; Final sample = 1807; Quality = Strong	Community (environmental and policy), Australia; 4-12 years; ≈ 50% girls	Reduce prevalence of childhood obesity	Nutrition and physical activity intervention; Prevention: Community capacity-building programme. Intervention included all manner of things. Targeted a variety of diet, physical activity and sedentary behaviours	**Universal:** No association between intervention effect and SES; SES associated with weight gain in control group	Waist circumference	↓	+
						BMI	↔	
						BMI z-score	↓	

^1^Global Quality appraisal from EPHPP (16); ^2^Prevention or treatment intervention; ^3^Targeted/Universal approach to inequality; ^4^p < 0.05.This is the relative mean differences between intervention and control at follow-up; ^5^+ positive intervention effect so it reduces obesity-related outcomes in low SES groups or reduces the SES gradient in obesity-related outcomes, 0 no intervention effect or no effect on SES gradient in obesity-related outcomes; *SES* = Socioeconomic status; *BMI* = Body mass index.

### Individual (n = 4 studies)

Three of the ‘best evidence’ studies included children of pre-school age (0-5 years), all of the studies included children of primary school age (6-12 years) and one study included children of secondary school age (13-18 years). Three of the studies were conducted in the USA and one in Australia. Study details are summarised in Table 1 with intervention effects sizes reported in Additional file 2: Tables S1-S2 and where data are available. None of the studies in this section were based in a school setting; study settings included healthcare centres, participant’s homes, and community sites. The studies included in this section have been grouped by a) those which aimed to prevent further weight gain in children at high risk of obesity, or treat obesity, b) those which aimed to prevent obesity, or improve obesity-related behaviours (diet and/or physical activity).

#### Interventions which aimed to prevent further weight gain in children at high obesity, or treat obesity (n = 3)

Two of the studies examined *tailored weight loss programmes* (face to face counselling on healthy diet and physical activity behaviours) delivered via primary care for boys and girls of all SES (universal approach). One was a cluster RCT [32] of 445 children aged 2-6 years conducted in the USA that found that following a one year intervention, there were no changes in BMI overall. However, BMI increased to a lesser extent in the intervention group compared with controls in children with household incomes of $50,000 or less (Additional file 2: Table S1). There was no intervention effect amongst children with household incomes greater than $50,000 or any differential effects by education status. A RCT [33] of 245 children aged 5-10 years conducted in Australia found that a 12 week intervention led to no significant differences between intervention and control groups for BMI, waist circumference, number overweight or obese at six or twelve months, and SES did not modify the intervention effect (Additional file 2: Table S1).

One RCT conducted in the USA investigated a *screen time reduction intervention* in 67 overweight children aged 4-7 of all SES (universal approach) [34]. Overall, there were greater reductions in BMI z scores over 24 months in the intervention group compared with controls (p < 0.05 for group x time interaction). In the low SES group there was a statistically significant between group difference for change in BMI z score at 6 months (mean difference between groups = -0.17; p = 0.002), 12 months (-0.20; p = 0.02), 18 months (-0.17; p = 0.04) and 24 months (-0.26; p = 0.05). There were no statistically significant between group differences in the high SES group.

#### Interventions which aimed to prevent obesity, or improve obesity-related behaviours (diet and/or physical activity) n = 1

The final individual study was a RCT [35] of an eleven week *mentor-based health promotion* in 179 black adolescents aged 11-16 years from low-income communities in the USA. After two years, there was no difference between intervention and control groups in change of BMI z score from baseline (Additional file 2: Table S2). However, the percentage of overweight and obese participants decreased in the intervention group (IG) compared with the control group (CG) (IG change = 45% to 39%; CG = 32% to 43%; p = 0.006). Overall, there were no between group differences in changes of percentage body fat, fat mass or fat free mass (Additional file 2: Table S2) but the intervention was effective at reducing percentage fat (β = -1.54; p = 0.003) and fat mass (β = -1.31; p = 0.025) and increasing fat-free mass (β = 1.41; p = 0.021) in participants who were overweight or obese.

### Community (n = 17 studies)

Half of the ‘best evidence’ community level studies (n = 7) were conducted in the USA with four from South American countries (two from Brazil, and one each from Chile and the Republic of Peru), four from Europe (two from the Netherlands and one each from Finland and France) and one each from Australia and Israel. The majority of studies (n = 13) included children of primary school age (6-12 years), three studies included children of preschool age (0-5 years) and one included adolescents (13-18 years). All of the studies included boys and girls (usually an approximately 50/50 mix), with the exception of three studies that included girls only. Study details are summarised in Table 2 with intervention effects sizes reported in Additional file 2: Tables S3 and S4 where data are available. The studies included in this section have been grouped, firstly, as school-based interventions or interventions in other settings, and then subdivided (where studies were available) into a) those which aimed to prevent obesity, or improve obesity-related behaviours (diet and/or physical activity) or b) those which aimed to prevent further weight gain in children at high risk of obesity, or treat obesity.

#### School-based interventions which aimed to prevent obesity, or improve obesity-related behaviours (diet and/or physical activity) n = 12

Twelve of the studies examined the effects on obesity related outcomes of *school based health promotion interventions*. Of these studies, nine investigated *nutrition and/or physical activity education combined with exercise sessions*; two studies examined *education only interventions* (diet and/or physical activity); and one study examined a *screen time reduction intervention*.

##### Nutrition and/or physical activity education combined with practical sessions (n = 9)

Three studies following a targeted approach found some positive results in terms of reducing obesity related indices in low SES children. The first was a non-randomised cluster controlled trial [36] of a six month *nutrition and physical activity education* intervention in low SES schools in Chile (n = 3084 children aged 11 years on average) that found positive intervention effects for boys in terms of BMI z score but not for BMI, triceps skinfold or waist circumference (Additional file 2: Table S4). No intervention effects were observed amongst girls (Additional file 2: Table S4). The second was a cluster randomised controlled trial [37] of an eight month diet and physical intervention in low income schools in the Netherlands (n = 2416 children aged 6-12 years). The intervention consisted of *exercise sessions* and *nutrition, physical activity and healthy lifestyle education*. In the younger children (6-9 years) there was no intervention effect for BMI; however, the increase in waist circumference was significantly smaller in the intervention group compared with the control (Additional file 2: Table S4). The prevalence of overweight in the intervention group also increased to a lesser extent compared to the controls. No intervention effects were observed amongst the older age group (Additional file 2: Table S4). The final study was a cluster RCT conducted in 11 kindergartens in low SES communities in Israel (n = 297 children aged 4-6 years) [38]. The intervention consisted of *nutrition education* and daily *exercise sessions*. Overall, greater decreases in BMI and BMI percentile were observed in the intervention group compared with controls (Additional file 2: Table S4); however, subgroup analysis revealed that this effect only occurred in boys and not girls.

A cluster RCT [39] that followed a universal approach investigated the effects of a cardiovascular disease risk factor reduction intervention delivered over eight weeks in 985 school children aged 8 to 10 years in the USA. Although there was a significant reduction in sum of skin folds from baseline to one year follow-up in the intervention group compared with controls (log of SSF mean change IG = -0.060; CG = -0.032; p = 0.0422), there was no relationship between intervention effects and SES of the children.

The final five studies found no beneficial intervention effects or any impact on inequalities in obesity. A randomised cluster trial [40] of a four-year school based multi-component *education and exercise* universal intervention to increase physical activity in 732 children aged 11-12 years in France (universal approach). At four year follow-up, intervention children showed a lower increase in age and gender adjusted BMI over time (p < 0.02), although there were no differential effects by SES. A cluster RCT [41] of an eighteen week targeted intervention in 201 low-income, minority pre-school children in the USA. The intervention comprised of *physical activity* and *nutrition education* sessions. There were no significant effects on BMI z score after 18 weeks. A non-randomised cluster controlled trial [42] investigated a school-based *physical activity education* and *exercise* targeted intervention (JUMP-in) among 2064 low SES 6-12 year olds in the Netherlands and found no intervention effects on BMI or waist circumference after 20 months (Additional file 2: Table S4). Another *physical activity education* and *exercise* session targeted intervention, delivered after-school, was investigated by a non-randomised cluster controlled trial [43] in a small sample of largely low-income children aged 10-11 years (n = 98) in the USA. No intervention effects in terms of change in BMI or BMI z score were observed after five months (Additional file 2: Table S4). A cluster RCT [44] among 294 adolescent girls (mean age = 13.2 years) in schools in low-income communities in Australia investigated the effects of a multi-component obesity prevention targeted programme that comprised of *nutrition and physical activity education* and *exercise* sessions. After 12 months there were no statistically significant intervention effects in terms of BMI, BMI z score or body fat change (Additional file 2: Table S4), although, there was a trend towards more beneficial changes in the intervention group for all of the outcomes.

##### Nutrition and/or physical activity education only (n = 2)

Two cluster RCTs examined *education only interventions* that were targeted at children aged 9-11 from low SES schools in the USA and Brazil. The Brazilian study was a cluster RCT [45] of an eight-month intervention to reduce sugar-sweetened beverage intake in schools that encouraged water consumption via competitions, promotions and provision of water bottles. It found no significant differences for all children (Additional file 2: Table S4), however, for girls – not boys - who were overweight at baseline there was a significant reduction of BMI in the intervention group (regression coefficient = -0.01; p = 0.009). However, the USA cluster RCT showed no intervention effect of a *diet and physical activity education only intervention* after one year [46] (Additional file 2: Table S4).

##### Screen time reduction only (n = 1)

A cluster randomised trial [47] investigated a *screen time reduction universal intervention* and showed beneficial effects in children aged 8 and 9 years after six months that were not associated with child SES. Post intervention, children in the intervention group had statistically significant relative reductions in: BMI (adjusted difference = -0.45 kg/m^2^ 95% CI -0.73 to -0.17, p = 0.002) as well as triceps skin fold thickness (adjusted difference = -1.47 mm, 95% CI -2.41 to -0.54, p = 0.002); waist circumference (adjusted difference = -2.30 cm, 95% CI -3.27 to -1.33, p < 0.001); and waist-hip ratio (adjusted difference = -0.02, 95% CI -0.03 to -0.01, p < 0.001). The results did not differ by SES.

##### Interventions in non-school settings which aimed to prevent further weight gain in children at high obesity, or treat obesity (n = 3)

Three of the ‘best evidence’ community level interventions evaluated *group-based weight loss programmes*. One was a RCT [48] of a six month *family-based education and behavioural therapy* universal programme compared with a standard treatment programme in 69 obese children aged 7-9 years in Finland of all SES. Beneficial intervention effects were observed: intervention children lost more weight for height than those receiving the routine treatment after six (IG 6.8% reduction; CG 1.8% reduction; p = 0.001) and twelve months (IG mean 3.4% reduction; CG mean 1.8% increase, p = 0.008) and there was a greater decrease in BMI in intervention children compared with routine treatment controls (IG change = -0.8, CG = 0.0, p = 0.003). However, there was no association between SES and outcomes (Additional file 2: Table S3).

The other two studies investigated *exercise based weight loss programmes* and found promising short term (<six months) results amongst primary school aged children from the USA and Brazil. A RCT [49] investigated the effects of a similar six month *exercise session targeted intervention* in 68 overweight children aged 5 to 10 years from a disadvantaged area in Brazil. After six months weight gain was less in the intervention group compared with controls (difference in change (IG-CG) = -1.37 kg; p < 0.001) and there was significant decrease in BMI in the intervention group compared with controls (difference in change (IG-CG) = -0.53 kg.m^2^; p = 0.049). A randomised controlled pilot study [50] investigated the effects of a twelve week *culturally appropriate exercise session and screen time reduction targeted intervention* amongst 61 low income African American girls aged 8 to 10 years in the USA. From baseline to post intervention, there were no significant differences between groups for changes in BMI and waist circumference (Additional file 2: Table S3); however, a trend towards better outcomes in the intervention group was noted.

##### Interventions in non-school settings which aimed to prevent obesity, or improve obesity-related behaviours (diet and/or physical activity) (n = 2)

The final two community level studies evaluated *group-based weight gain prevention educational targeted interventions* in low SES children (one targeted parents only). These studies found that the interventions did not lead to beneficial effects after a relatively long follow-up (one year) in pre-school and primary school aged children. One was a non-randomised controlled study [51] of the effects of a mother and daughter twelve week *culturally specific group based weight gain prevention educational intervention* amongst 40 low SES, African American girls aged 7 to 12 years in USA (mean age = 10.0 years). No intervention effects were observed for obesity outcomes after one year follow-up (Additional 2: Table S3). The other study was a RCT [52] of a *health education intervention* delivered to 1501 microcredit clients (families too poor to borrow from traditional lending institutions) in addition to their loans on their children aged less than five years in the Republic of Peru. The health education intervention was delivered by trained loan officers over eight months and covered basic child health provision, and discussion of clients own experiences and problem solving. There were no differences between the control and intervention groups in the change in the percentage of children who were overweight and in mean BMI z scores from baseline to one follow-up (Additional file 2: Table S3).

### Societal (n = 1 study)

The ‘best available’ evidence for the environmental interventions comes from one strong quality experimental study that followed a universal approach and compared intervention effects of low SES children (using parent education as a proxy measure) versus higher SES children [53, 54]. The study examined a *multi-faceted school based obesity prevention intervention* that was conducted in pre-schools in Switzerland*.* The intervention included changes to the school’s environment to encourage physical activity (fixed and mobile equipment such as climbing walls, hammocks, balls and stilts) along with the provision of healthy snacks, nutrition education and exercise sessions. This reasonably sized study (n = 625) found some beneficial intervention effects overall after 9½ months in terms of body fat and waist circumference, but not BMI or prevalence of overweight [54]. Sub-group analysis revealed no significant differences in intervention effects between children with low education parents and those with parents of medium/high education; however, there was a trend towards more beneficial effects in the higher SES children [53].

### Mixed - individual, community and societal (n = 1 study)

One strong quality experimental study [55] examined the effects of a three year *community capacity-building intervention* amongst 1,807 children of all SES aged 4-12 years in Australia (universal approach). The intervention was designed by a number of key organisations to build the community’s capacity to create its own solutions to promoting healthy eating, physical activity and healthy weight, and the delivered universally in all intervention schools. After three years, children in the intervention schools showed significantly lower increases in waist circumference (adjusted difference between comparison and intervention = -3.14; p = 0.01) and BMI z-score (adjusted difference = -0.11; p = 0.04) compared with the children in the control schools. There was no association between SES measures and intervention effects in the intervention schools; however, lower SES was associated with a greater gain in body fat and waist circumference in the control schools. Therefore, the intervention halted the widening of inequalities in obesity that would normally naturally occur over time.

## Discussion

### What works in reducing inequalities in obesity-related outcomes? for whom? and where?

At an individual level, the results from the ‘best available’ evidence (n = 4) identified by our review suggests that *mentor-based health promotion interventions* may be effective in reducing obesity prevalence in low SES children as there were particular benefits to those low income children who were already overweight or obese (one year); and that a *screen time reduction intervention* was more beneficial for low SES children after two years.

The ‘best available’ evidence of the effectiveness of community level interventions (n = 17) was mixed, as whilst some studies identified effective interventions both in the short- and long-term (amongst children aged 6-12), others did not. Therefore, this review has found that there is some – but not conclusive evidence – that *school based nutrition and physical activity education and exercise sessions* and *school-delivered screen time reduction interventions* were effective in the longer term (over six months) in reducing obesity-related outcomes amongst school aged children with no differential effects by SES. In the shorter-term (up to six months), *family based education and behavioural weight loss programmes,* and *exercise based weight loss programmes* targeted at low SES school aged children were effective in reducing obesity-related outcomes. There was some evidence of effectiveness of *school based nutrition and physical activity education and exercise sessions* amongst pre-school children in the longer-term (one year). There was no evidence of effectiveness from the one ‘best evidence’ study amongst adolescents.

Our review identified only one strong quality experimental study of more upstream environmental interventions and no studies of the effects of macro-level policy interventions on obesity-related outcomes amongst children. The *multi-faceted school based obesity prevention intervention* was found to be effective amongst pre-school children in the longer term (over six months) but with slightly more beneficial effects to those of higher SES. Finally, a multi-level *community capacity-building intervention* was effective in preventing a widening of inequalities in obesity amongst children aged 4-12 over the long term (up to three years).

The majority of the ‘best available’ evidence was from interventions conducted in the USA or South America. In most cases interventions appeared to be equally effective – or ineffective - for boys and girls – although some studies did not distinguish their results by gender. Most of the studies were of interventions targeted at low SES children/areas and often of ‘treatment’ interventions for those already overweight or obese. In terms of ‘where’ interventions appeared to be effective, the ‘best available’ evidence was dominated by school-delivered interventions and this suggested that school-based interventions targeted at low SES children could have some beneficial effects in reducing inequalities – although the evidence was by no means conclusive. This is in line with the ‘Whole School Approach’ to tackling childhood obesity. The findings of effectiveness are therefore very much limited to the effectiveness of school-based interventions, for low-income, primary school-aged children (6-12 years), particularly in the USA.

We did try to further unpick the various programme components that were used in the studies included in this review, to identify in detail why some interventions had a positive impact on inequalities in obesity-related outcomes, and others did not, particularly for interventions which appeared similar but had different effects. We did not systematically look for separate process evaluations as this was beyond the scope of the review, although we acknowledge that would have been helpful. From the information provided to us in the research papers, there were no consistent themes which shone through beyond those mentioned above. Importantly, there was no evidence that the studies included in this review increased inequalities in obesity-related outcomes. Whilst we wait for results of ongoing relevant interventions, and assuming interventions which tackle obesity-related outcomes do not increase inequalities, we suggest that interested stakeholders refer to a Cochrane review by Waters [30] which suggest the following to be promising policies and strategies:

- school curriculum that includes healthy eating, physical activity and body image
- increased sessions for physical activity and the development of fundamental movement skills throughout the school week
- improvements in nutritional quality of the food supply in schools
- environments and cultural practices that support children eating healthier foods and being active throughout each day
- support for teachers and other staff to implement health promotion strategies and activities (e.g. professional development, capacity building activities)
- parent support and home activities that encourage children to be more active, eat more nutritious foods and spend less time in screen based activities

### Implications for research

The direction of research and evaluation in this field must move into how to implement effectively to scale, sustain the impacts over time, and ensure equitable outcomes of interventions to manage childhood obesity and reduce associated inequalities. We recommend larger, longer term studies, powered to detect the small changes that are likely to be found, with assessments of equity impacts, to enable translation of research findings into effective public health approaches for managing childhood obesity.

The majority of interventions that we included in this part of our review were aimed at preventing weight gain, although a number of ‘treatment’ interventions were also included. These ‘treatment’ interventions are more likely to show positive effects than prevention ones. The targeted approach also has limitations as even when interventions are effective amongst low income groups they are only able to reduce the health inequalities gap, they have little effect on the wider social gradient. Most studies were school based and aimed at primary school aged children (6-12 years). There were also very few studies of societal level interventions that might be expected to have more of an impact on the gradient in obesity [27]. We also found no studies that assessed the cost-effectiveness of interventions and meta-analysis was not appropriate given their heterogeneity.

Our results show that there is a clear need for more experimental studies of the effectiveness and cost-effectiveness of interventions to reduce inequalities in childhood obesity, particularly in adolescents and in terms of macro-level interventions that potentially address the entire gradient. There has been a real missed opportunity to evaluate the effects of such ‘real world’ interventions, and future interventions (such as Fulfilling Lives: A Better Start) should include such analysis. It is worth highlighting the ongoing EPHE (EPODE for the Promotion of Health Equity) evaluation study which aims to provide useful information about the impact of the EPODE intervention on socioeconomic inequalities across Europe; ‘**E**nsemble **P**révenons l’**O**bésité **D**es **E**nfants’ (EPODE, *Together Let’s Prevent Childhood Obesity*) is a large-scale, coordinated, capacity-building approach for communities to implement effective and sustainable strategies to prevent childhood obesity [56].

### Implications for public health

Our review has found only limited effectiveness of interventions with the potential to reduce SES inequalities in obesity. The body of evidence in this review provides some support for the hypothesis that obesity treatment interventions in children can be effective and that for interventions targeted at low SES children they have reduced obesity-related outcomes; for universal interventions they have reduced the SES gradient in obesity-related outcomes. Interventions need to be developed so that they can be embedded into ongoing practice and operating systems, rather than implementing interventions that are resource intensive and cannot be maintained long-term. This review also highlights that although we may now have a good sense of the range of interventions feasible for use in reducing the risk of childhood obesity, we lack the knowledge of which specific intervention components are most effective to ensure that the equity gradients reduced. Being able to answer this question is of critical importance to decision makers.

### Strengths and limitations

This review was very extensive as an extremely thorough search was conducted of the international literature with a very broad inclusion and exclusion criteria that has ensured that the entire relevant evidence base was captured. This has enabled us to focus in this paper on just the best available (experimental) evidence. Quality is additionally high as double screening was applied and both data extraction and quality appraisal were independently checked. However, the review is still subject to some methodological limitations as for example the quality assessment tool, although described as a tool for public health interventions, seemed to favour those that followed a more clinical model. We particularly found the blinding question unhelpful as it mostly resulted in moderate scores. The definitions for level of intervention that were used, adapted from the health inequalities literature, meant that most studies were categorised as community level interventions. Other ways of categorising studies (such as by whether primary prevention programmes are more/less/even effective in decreasing or haltering inequalities compared to selective prevention programmes), or by examining the theoretical underpinning of interventions (such as those that are based on Social Cognitive Theory, Planned Behaviour or theories of Environment–Behaviour Relationships) could also have been used. One final limitation is our exclusion of studies that examined ethnic inequalities that may have reduced the USA literature where ethnicity is often used as a proxy for SES.

## Conclusion

Our review has found only limited evidence of the effectiveness of interventions with the potential to reduce SES inequalities in obesity-related outcomes amongst children. These findings suggest that individual, community and societal-level interventions that aim to prevent obesity, treat obesity, or improve obesity-related behaviours (diet and/or physical activity) do not increase socioeconomic inequalities; many of the universal interventions have the potential to slow the widening of the obesity gap, and some of the interventions which are targeted at low SES children may be effective in decreasing obesity amongst lower socio-economic groups. Experimental studies of the effectiveness and cost-effectiveness of interventions to reduce inequalities in childhood obesity are needed, particularly in adolescents and in terms of macro-level interventions that potentially address the entire gradient, as well as evaluations of ‘real world’ interventions.

## Electronic supplementary material

Additional file 1: **Search Strategy – MEDLINE (Ovid).** (DOC 102 KB)

Additional file 2: **Effect size tables.** **Table S1.** Intervention effect sizes of the individual level intervention, universal experimental studies. **Table S2.** Intervention effect sizes of the individual level intervention, targeted (disadvantaged groups only) experimental studies. **Table S3.** Intervention effect sizes of the community level intervention, universal experimental studies. **Table S4.** Intervention effect sizes of the community level intervention, targeted (disadvantaged groups only) experimental studies. (DOCX 22 KB)
