# Supplementary material for: A systematic review of the effectiveness of individual, community and societal level interventions at reducing socioeconomic inequalities in obesity amongst children
Source: BMC Public Health. 2014 Aug 11;14:834. doi: 10.1186/1471-2458-14-834 (PMC4137097; doi:10.1186/1471-2458-14-834)
Supplement: Supplementary file 1 — Additional file 1: Search Strategy – MEDLINE (Ovid). (DOC 102 KB) [file 12889_2014_6950_MOESM1_ESM.doc]

**Search Strategy – MEDLINE (Ovid)**

| 1 | "Body Weights and Measures"/ |
| --- | --- |
| 2 | (BMI or Body Mass Index).ti,ab. or Body Weight/ or obesity.ti,ab. or obese.ti,ab. or overweight.ti,ab. or weight gain.ti,ab. or weight loss.ti,ab. or exp OBESITY/ or Body fat.ti,ab. or Fat mass.ti,ab. or Weight control$.ti,ab. or Weight maintain$.ti,ab. or Adipos$.ti,ab. or Adipose tissue.ti,ab. or Skinfold thickness.ti,ab. or Waist circumference.ti,ab. or Waist hip ratio.ti,ab. or WHR.ti,ab. |
| 3 | 1 or 2 |
| 4 | Health Promotion/ or health promotion.ti,ab. or health behaviour.ti,ab. or health behavior.ti,ab. or (policy and (social or school or food or public or urban or environmental or fiscal)).ti,ab. or urban planning.ti,ab. or city planning.ti,ab. or built environment.ti,ab. or social environment.ti,ab. or physical environment.ti,ab. or cultural environment.ti,ab. or urban environment.ti,ab. or school environment.ti,ab. or neighbourhood.ti,ab. or community.ti,ab. or societal.ti,ab. or social interventions.ti,ab. or community interventions.ti,ab. or obesogenic environment.ti,ab. or individual level.ti,ab. or lifestyle.ti,ab. or individual.ti,ab. or tax$.ti,ab. or subsid$.ti,ab. or price$.ti,ab. or health education.ti,ab. or social marketing.ti,ab. or (diet and (advice or counselling)).ti,ab. or (exercise and (advice or counselling)).ti,ab. or weight management.ti,ab. or cash transfer$.ti,ab. or lifestyle counselling.ti,ab. or behavioural counselling.ti,ab. or behavioral counselling.ti,ab. or exercise on prescription.ti,ab. or exercise.ti,ab. or health trainer$.ti,ab. or school.ti,ab. or workplace.ti,ab. or campaign$.ti,ab. or (access adj1 facilities).ti,ab. or green space.ti,ab. or walk?ability.ti,ab. or food label$.ti,ab. or food advert$.ti,ab. |
| 5 | (evaluat$ or effective$ or Intervention or RCT or experiment$ or randomi?ed controlled trial$ or clinical randomi?ed controlled trial$ or cluster randomi?ed controlled trial$ or double blind randomi?ed controlled trial$ or randomi?ed consent design or single blind randomi?ed controlled trial$ or randomi?ed or placebo or random$ or trial or quasi?experiment$ or pre$test or post$test or trial or time series or evaluat$ or intervention$ or "before and after" or intervention$ or community trial or non?randomi?ed or repeat$ or repeat$ measures).ti,ab. or (exp Clinical Trial/ or exp Randomized Controlled Trial/ or exp Randomization/ or exp Double-Blind Method/ or exp Single-Blind Method/ or exp Cross-Over Studies/) or clinical trial.ti,ab. or latin square.ti,ab. or random$.ti,ab. or exp Evaluation/ or clinical trial.ti,ab. or clinical trial.pt. or (before adj1 after adj1 (stud$ or trial$ or design$)).ti,ab. or random$.ti,ab. or (quasi?experimental or pseudo?experimental).ti,ab. or (nonrandomi?ed or non?randomi?ed or pseudo?randomi?sed or quasi?randomi?ed).ti,ab. or ((population level or population based or population orientated or population oriented or community level or community based or community orientated or community oriented) adj3 (intervention$ or prevention or policy or policies or program$ or project$)).ti,ab. |
| 6 | 3 and 4 and 5 |
| 7 | limit 6 to humans |

**Embase Search Ovid)**

| 1 | "Body Weights and Measures"/ |
| --- | --- |
| 2 | (BMI or Body Mass Index).ti,ab. or Body Weight/ or obesity.ti,ab. or obese.ti,ab. or overweight.ti,ab. or weight gain.ti,ab. or weight loss.ti,ab. or exp OBESITY/ or Body fat.ti,ab. or Fat mass.ti,ab. or Weight control$.ti,ab. or Weight maintain$.ti,ab. or Adipos$.ti,ab. or Adipose tissue.ti,ab. or Skinfold thickness.ti,ab. or Waist circumference.ti,ab. or Waist hip ratio.ti,ab. or WHR.ti,ab. |
| 3 | 1 or 2 |
| 4 | Health Promotion/ or health promotion.ti,ab. or health behaviour.ti,ab. or health behavior.ti,ab. or (policy and (social or school or food or public or urban or environmental or fiscal)).ti,ab. or urban planning.ti,ab. or city planning.ti,ab. or built environment.ti,ab. or social environment.ti,ab. or physical environment.ti,ab. or cultural environment.ti,ab. or urban environment.ti,ab. or school environment.ti,ab. or neighbourhood.ti,ab. or community.ti,ab. or societal.ti,ab. or social interventions.ti,ab. or community interventions.ti,ab. or obesogenic environment.ti,ab. or individual level.ti,ab. or lifestyle.ti,ab. or individual.ti,ab. or tax$.ti,ab. or subsid$.ti,ab. or price$.ti,ab. or health education.ti,ab. or social marketing.ti,ab. or (diet and (advice or counselling)).ti,ab. or (exercise and (advice or counselling)).ti,ab. or weight management.ti,ab. or cash transfer$.ti,ab. or lifestyle counselling.ti,ab. or behavioural counselling.ti,ab. or behavioral counselling.ti,ab. or exercise on prescription.ti,ab. or exercise.ti,ab. or health trainer$.ti,ab. or school.ti,ab. or workplace.ti,ab. or campaign$.ti,ab. or (access adj1 facilities).ti,ab. or green space.ti,ab. or walk?ability.ti,ab. or food label$.ti,ab. or food advert$.ti,ab. |
| 5 | (evaluat$ or effective$ or Intervention or RCT or experiment$ or randomi?ed controlled trial$ or clinical randomi?ed controlled trial$ or cluster randomi?ed controlled trial$ or double blind randomi?ed controlled trial$ or randomi?ed consent design or single blind randomi?ed controlled trial$ or randomi?ed or placebo or random$ or trial or quasi?experiment$ or pre$test or post$test or trial or time series or evaluat$ or intervention$ or "before and after" or intervention$ or community trial or non?randomi?ed or repeat$ or repeat$ measures).ti,ab. or (exp Clinical Trial/ or exp Randomized Controlled Trial/ or exp Randomization/ or exp Double-Blind Method/ or exp Single-Blind Method/ or exp Cross-Over Studies/) or clinical trial.ti,ab. or latin square.ti,ab. or random$.ti,ab. or exp Evaluation/ or clinical trial.ti,ab. or clinical trial.pt. or (before adj1 after adj1 (stud$ or trial$ or design$)).ti,ab. or random$.ti,ab. or (quasi?experimental or pseudo?experimental).ti,ab. or (nonrandomi?ed or non?randomi?ed or pseudo?randomi?sed or quasi?randomi?ed).ti,ab. or ((population level or population based or population orientated or population oriented or community level or community based or community orientated or community oriented) adj3 (intervention$ or prevention or policy or policies or program$ or project$)).ti,ab. |
| 6 | 3 and 4 and 5 |
| 7 | limit 6 to humans |
| 8 | limit 7 to last year |

**IBSS Search Proquest)**

| **S5** | all((BMI OR Body Mass Index) OR (obesity) OR (obese) OR (overweight) OR (weight gain) OR (weight loss) OR (Body fat) OR (Fat mass) OR (Weight control*) OR (Weight maintain*) OR (Adipos*) OR (Adipose tissue) OR (Skinfold thickness) OR (Waist circumference) OR (Waist hip ratio) OR (WHR)) AND all((health promotion) OR (health behaviour) OR (health behavior) OR (policy AND (social OR school OR food OR public OR urban OR environmental OR fiscal)) OR (urban planning) OR (city planning) OR (built environment) OR (social environment) OR (physical environment) OR (cultural environment) OR (urban environment) OR (school environment) OR (neighbourhood) OR (community) OR (societal) OR (social interventions) OR (community interventions) OR (obesogenic environment) OR (individual level) OR (lifestyle) OR (individual) OR (tax*) OR (subsid*) OR (price*) OR (health education) OR (social marketing) OR (diet AND (advice OR counselling)) OR (exercise AND (advice OR counselling)) OR (weight management) OR (cash transfer*) OR (lifestyle counselling) OR (behavioural counselling) OR (behavioral counselling) OR (exercise on prescription) OR (exercise) OR (health trainer*) OR (school) OR (workplace) OR (campaign*) OR (access NEAR/1 facilities) OR (green space) OR (walk*ability) OR (food label*) OR (food advert*)) AND ((evaluat* or effective* or Intervention or RCT or experiment* or randomi?ed controlled trial* or clinical randomi?ed controlled trial* or cluster randomi?ed controlled trial* or double blind randomi?ed controlled trial* or randomi?ed consent design or single blind randomi?ed controlled trial* or randomi?ed or placebo or random* or trial or quasi?experiment* or pre*test or post*test or trial or time series or evaluat* or intervention* or "before and after" or intervention* or community trial or non?randomi?ed or repeat* or repeat* measures) or (clinical trial or latin square or random* or clinical trial) or (before NEAR/1 after NEAR/1 (stud* or trial* or design*)) or random* or (quasi?experimental or pseudo?experimental) or (nonrandomi?ed or non?randomi?ed or pseudo?randomi?sed or quasi?randomi?ed) or (population level or population based or population orientated or population oriented or community level or community based or community orientated or community oriented) or (intervention* or prevention or policy or policies or program* or project*))Limits applied  **Databases:**  **Narrowed by:**  Entered date: 10/ 2011 - 10/ 2012 |
| --- | --- |
| **S4** | all((BMI OR Body Mass Index) OR (obesity) OR (obese) OR (overweight) OR (weight gain) OR (weight loss) OR (Body fat) OR (Fat mass) OR (Weight control*) OR (Weight maintain*) OR (Adipos*) OR (Adipose tissue) OR (Skinfold thickness) OR (Waist circumference) OR (Waist hip ratio) OR (WHR)) AND all((health promotion) OR (health behaviour) OR (health behavior) OR (policy AND (social OR school OR food OR public OR urban OR environmental OR fiscal)) OR (urban planning) OR (city planning) OR (built environment) OR (social environment) OR (physical environment) OR (cultural environment) OR (urban environment) OR (school environment) OR (neighbourhood) OR (community) OR (societal) OR (social interventions) OR (community interventions) OR (obesogenic environment) OR (individual level) OR (lifestyle) OR (individual) OR (tax*) OR (subsid*) OR (price*) OR (health education) OR (social marketing) OR (diet AND (advice OR counselling)) OR (exercise AND (advice OR counselling)) OR (weight management) OR (cash transfer*) OR (lifestyle counselling) OR (behavioural counselling) OR (behavioral counselling) OR (exercise on prescription) OR (exercise) OR (health trainer*) OR (school) OR (workplace) OR (campaign*) OR (access NEAR/1 facilities) OR (green space) OR (walk*ability) OR (food label*) OR (food advert*)) AND ((evaluat* or effective* or Intervention or RCT or experiment* or randomi?ed controlled trial* or clinical randomi?ed controlled trial* or cluster randomi?ed controlled trial* or double blind randomi?ed controlled trial* or randomi?ed consent design or single blind randomi?ed controlled trial* or randomi?ed or placebo or random* or trial or quasi?experiment* or pre*test or post*test or trial or time series or evaluat* or intervention* or "before and after" or intervention* or community trial or non?randomi?ed or repeat* or repeat* measures) or (clinical trial or latin square or random* or clinical trial) or (before NEAR/1 after NEAR/1 (stud* or trial* or design*)) or random* or (quasi?experimental or pseudo?experimental) or (nonrandomi?ed or non?randomi?ed or pseudo?randomi?sed or quasi?randomi?ed) or (population level or population based or population orientated or population oriented or community level or community based or community orientated or community oriented) or (intervention* or prevention or policy or policies or program* or project*))  **Databases:** |
| **S3** | (evaluat* or effective* or Intervention or RCT or experiment* or randomi?ed controlled trial* or clinical randomi?ed controlled trial* or cluster randomi?ed controlled trial* or double blind randomi?ed controlled trial* or randomi?ed consent design or single blind randomi?ed controlled trial* or randomi?ed or placebo or random* or trial or quasi?experiment* or pre*test or post*test or trial or time series or evaluat* or intervention* or "before and after" or intervention* or community trial or non?randomi?ed or repeat* or repeat* measures) or (clinical trial or latin square or random* or clinical trial) or (before NEAR/1 after NEAR/1 (stud* or trial* or design*)) or random* or (quasi?experimental or pseudo?experimental) or (nonrandomi?ed or non?randomi?ed or pseudo?randomi?sed or quasi?randomi?ed) or (population level or population based or population orientated or population oriented or community level or community based or community orientated or community oriented) or (intervention* or prevention or policy or policies or program* or project*)  **Databases:** |
| **S2** | all((health promotion) or (health behaviour) or (health behavior) or (policy and (social or school or food or public or urban or environmental or fiscal)) or (urban planning) or (city planning) or (built environment) or (social environment) or (physical environment) or (cultural environment) or (urban environment) or (school environment) or (neighbourhood) or (community) or (societal) or (social interventions) or (community interventions) or (obesogenic environment) or (individual level) or (lifestyle) or (individual) or (tax*) or (subsid*) or (price*) or (health education) or (social marketing) or (diet and (advice or counselling)) or (exercise and (advice or counselling)) or (weight management) or (cash transfer*) or (lifestyle counselling) or (behavioural counselling) or (behavioral counselling) or (exercise on prescription) or (exercise) or (health trainer*) or (school) or (workplace) or (campaign*) or (access NEAR/1 facilities) or (green space) or (walk*ability) or (food label*) or (food advert*))  **Databases:** |
| **S1** | all((BMI or Body Mass Index) or (obesity) or (obese) or (overweight) or (weight gain) or (weight loss) or (Body fat) or (Fat mass) or (Weight control*) or (Weight maintain*) or (Adipos*) or (Adipose tissue) or (Skinfold thickness) or (Waist circumference) or (Waist hip ratio) or (WHR)) |

**ASSIA (Proquest)**

| **S5** | ((BMI or Body Mass Index) or (obesity) or (obese) or (overweight) or (weight gain) or (weight loss) or (Body fat) or (Fat mass) or (Weight control*) or (Weight maintain*) or (Adipos*) or (Adipose tissue) or (Skinfold thickness) or (Waist circumference) or (Waist hip ratio) or (WHR)) AND ((health promotion) or (health behaviour) or (health behavior) or (policy and (social or school or food or public or urban or environmental or fiscal)) or (urban planning) or (city planning) or (built environment) or (social environment) or (physical environment) or (cultural environment) or (urban environment) or (school environment) or (neighbourhood) or (community) or (societal) or (social interventions) or (community interventions) or (obesogenic environment) or (individual level) or (lifestyle) or (individual) or (tax*) or (subsid*) or (price*) or (health education) or (social marketing) or (diet and (advice or counselling)) or (exercise and (advice or counselling)) or (weight management) or (cash transfer*) or (lifestyle counselling) or (behavioural counselling) or (behavioral counselling) or (exercise on prescription) or (exercise) or (health trainer*) or (school) or (workplace) or (campaign*) or (access NEAR/1 facilities) or (green space) or (walk*ability) or (food label*) or (food advert*)) AND ((evaluat* or effective* or Intervention or RCT or experiment* or randomi?ed controlled trial* or clinical randomi?ed controlled trial* or cluster randomi?ed controlled trial* or double blind randomi?ed controlled trial* or randomi?ed consent design or single blind randomi?ed controlled trial* or randomi?ed or placebo or random* or trial or quasi?experiment* or pre*test or post*test or trial or time series or evaluat* or intervention* or "before and after" or intervention* or community trial or non?randomi?ed or repeat* or repeat* measures) or (clinical trial or latin square or random* or clinical trial) or (before NEAR/1 after NEAR/1 (stud* or trial* or design*)) or random* or (quasi?experimental or pseudo?experimental) or (nonrandomi?ed or non?randomi?ed or pseudo?randomi?sed or quasi?randomi?ed) or (population level or population based or population orientated or population oriented or community level or community based or community orientated or community oriented) or (intervention* or prevention or policy or policies or program* or project*))Limits applied**Databases:**  **Narrowed by:**  Entered date: 10/ 2011 - 10/ 2012 |
| --- | --- |
| **S4** | ((BMI or Body Mass Index) or (obesity) or (obese) or (overweight) or (weight gain) or (weight loss) or (Body fat) or (Fat mass) or (Weight control*) or (Weight maintain*) or (Adipos*) or (Adipose tissue) or (Skinfold thickness) or (Waist circumference) or (Waist hip ratio) or (WHR)) AND ((health promotion) or (health behaviour) or (health behavior) or (policy and (social or school or food or public or urban or environmental or fiscal)) or (urban planning) or (city planning) or (built environment) or (social environment) or (physical environment) or (cultural environment) or (urban environment) or (school environment) or (neighbourhood) or (community) or (societal) or (social interventions) or (community interventions) or (obesogenic environment) or (individual level) or (lifestyle) or (individual) or (tax*) or (subsid*) or (price*) or (health education) or (social marketing) or (diet and (advice or counselling)) or (exercise and (advice or counselling)) or (weight management) or (cash transfer*) or (lifestyle counselling) or (behavioural counselling) or (behavioral counselling) or (exercise on prescription) or (exercise) or (health trainer*) or (school) or (workplace) or (campaign*) or (access NEAR/1 facilities) or (green space) or (walk*ability) or (food label*) or (food advert*)) AND ((evaluat* or effective* or Intervention or RCT or experiment* or randomi?ed controlled trial* or clinical randomi?ed controlled trial* or cluster randomi?ed controlled trial* or double blind randomi?ed controlled trial* or randomi?ed consent design or single blind randomi?ed controlled trial* or randomi?ed or placebo or random* or trial or quasi?experiment* or pre*test or post*test or trial or time series or evaluat* or intervention* or "before and after" or intervention* or community trial or non?randomi?ed or repeat* or repeat* measures) or (clinical trial or latin square or random* or clinical trial) or (before NEAR/1 after NEAR/1 (stud* or trial* or design*)) or random* or (quasi?experimental or pseudo?experimental) or (nonrandomi?ed or non?randomi?ed or pseudo?randomi?sed or quasi?randomi?ed) or (population level or population based or population orientated or population oriented or community level or community based or community orientated or community oriented) or (intervention* or prevention or policy or policies or program* or project*))  **Databases:** |
| **S3** | (evaluat* or effective* or Intervention or RCT or experiment* or randomi?ed controlled trial* or clinical randomi?ed controlled trial* or cluster randomi?ed controlled trial* or double blind randomi?ed controlled trial* or randomi?ed consent design or single blind randomi?ed controlled trial* or randomi?ed or placebo or random* or trial or quasi?experiment* or pre*test or post*test or trial or time series or evaluat* or intervention* or "before and after" or intervention* or community trial or non?randomi?ed or repeat* or repeat* measures) or (clinical trial or latin square or random* or clinical trial) or (before NEAR/1 after NEAR/1 (stud* or trial* or design*)) or random* or (quasi?experimental or pseudo?experimental) or (nonrandomi?ed or non?randomi?ed or pseudo?randomi?sed or quasi?randomi?ed) or (population level or population based or population orientated or population oriented or community level or community based or community orientated or community oriented) or (intervention* or prevention or policy or policies or program* or project*)  **Databases:** |
| **S2** | (health promotion) or (health behaviour) or (health behavior) or (policy and (social or school or food or public or urban or environmental or fiscal)) or (urban planning) or (city planning) or (built environment) or (social environment) or (physical environment) or (cultural environment) or (urban environment) or (school environment) or (neighbourhood) or (community) or (societal) or (social interventions) or (community interventions) or (obesogenic environment) or (individual level) or (lifestyle) or (individual) or (tax*) or (subsid*) or (price*) or (health education) or (social marketing) or (diet and (advice or counselling)) or (exercise and (advice or counselling)) or (weight management) or (cash transfer*) or (lifestyle counselling) or (behavioural counselling) or (behavioral counselling) or (exercise on prescription) or (exercise) or (health trainer*) or (school) or (workplace) or (campaign*) or (access NEAR/1 facilities) or (green space) or (walk*ability) or (food label*) or (food advert*)  **Databases:** |
| **S1** | (BMI or Body Mass Index) or (obesity) or (obese) or (overweight) or (weight gain) or (weight loss) or (Body fat) or (Fat mass) or (Weight control*) or (Weight maintain*) or (Adipos*) or (Adipose tissue) or (Skinfold thickness) or (Waist circumference) or (Waist hip ratio) or (WHR)  **Databases:** |

**Sociological Abstracts (Proquest)**

| **S5** | ((BMI or Body Mass Index) or (obesity) or (obese) or (overweight) or (weight gain) or (weight loss) or (Body fat) or (Fat mass) or (Weight control*) or (Weight maintain*) or (Adipos*) or (Adipose tissue) or (Skinfold thickness) or (Waist circumference) or (Waist hip ratio) or (WHR)) AND ((health promotion) or (health behaviour) or (health behavior) or (policy and (social or school or food or public or urban or environmental or fiscal)) or (urban planning) or (city planning) or (built environment) or (social environment) or (physical environment) or (cultural environment) or (urban environment) or (school environment) or (neighbourhood) or (community) or (societal) or (social interventions) or (community interventions) or (obesogenic environment) or (individual level) or (lifestyle) or (individual) or (tax*) or (subsid*) or (price*) or (health education) or (social marketing) or (diet and (advice or counselling)) or (exercise and (advice or counselling)) or (weight management) or (cash transfer*) or (lifestyle counselling) or (behavioural counselling) or (behavioral counselling) or (exercise on prescription) or (exercise) or (health trainer*) or (school) or (workplace) or (campaign*) or (access NEAR/1 facilities) or (green space) or (walk*ability) or (food label*) or (food advert*)) AND ((evaluat* or effective* or Intervention or RCT or experiment* or randomi?ed controlled trial* or clinical randomi?ed controlled trial* or cluster randomi?ed controlled trial* or double blind randomi?ed controlled trial* or randomi?ed consent design or single blind randomi?ed controlled trial* or randomi?ed or placebo or random* or trial or quasi?experiment* or pre*test or post*test or trial or time series or evaluat* or intervention* or "before and after" or intervention* or community trial or non?randomi?ed or repeat* or repeat* measures) or (clinical trial or latin square or random* or clinical trial) or (before NEAR/1 after NEAR/1 (stud* or trial* or design*)) or random* or (quasi?experimental or pseudo?experimental) or (nonrandomi?ed or non?randomi?ed or pseudo?randomi?sed or quasi?randomi?ed) or (population level or population based or population orientated or population oriented or community level or community based or community orientated or community oriented) or (intervention* or prevention or policy or policies or program* or project*))Limits applied**Databases:**  **Narrowed by:**  Entered date: 10/ 2011 - 10/ 2012 |
| --- | --- |
| **S4** | ((BMI or Body Mass Index) or (obesity) or (obese) or (overweight) or (weight gain) or (weight loss) or (Body fat) or (Fat mass) or (Weight control*) or (Weight maintain*) or (Adipos*) or (Adipose tissue) or (Skinfold thickness) or (Waist circumference) or (Waist hip ratio) or (WHR)) AND ((health promotion) or (health behaviour) or (health behavior) or (policy and (social or school or food or public or urban or environmental or fiscal)) or (urban planning) or (city planning) or (built environment) or (social environment) or (physical environment) or (cultural environment) or (urban environment) or (school environment) or (neighbourhood) or (community) or (societal) or (social interventions) or (community interventions) or (obesogenic environment) or (individual level) or (lifestyle) or (individual) or (tax*) or (subsid*) or (price*) or (health education) or (social marketing) or (diet and (advice or counselling)) or (exercise and (advice or counselling)) or (weight management) or (cash transfer*) or (lifestyle counselling) or (behavioural counselling) or (behavioral counselling) or (exercise on prescription) or (exercise) or (health trainer*) or (school) or (workplace) or (campaign*) or (access NEAR/1 facilities) or (green space) or (walk*ability) or (food label*) or (food advert*)) AND ((evaluat* or effective* or Intervention or RCT or experiment* or randomi?ed controlled trial* or clinical randomi?ed controlled trial* or cluster randomi?ed controlled trial* or double blind randomi?ed controlled trial* or randomi?ed consent design or single blind randomi?ed controlled trial* or randomi?ed or placebo or random* or trial or quasi?experiment* or pre*test or post*test or trial or time series or evaluat* or intervention* or "before and after" or intervention* or community trial or non?randomi?ed or repeat* or repeat* measures) or (clinical trial or latin square or random* or clinical trial) or (before NEAR/1 after NEAR/1 (stud* or trial* or design*)) or random* or (quasi?experimental or pseudo?experimental) or (nonrandomi?ed or non?randomi?ed or pseudo?randomi?sed or quasi?randomi?ed) or (population level or population based or population orientated or population oriented or community level or community based or community orientated or community oriented) or (intervention* or prevention or policy or policies or program* or project*))  **Databases:** |
| **S3** | (evaluat* or effective* or Intervention or RCT or experiment* or randomi?ed controlled trial* or clinical randomi?ed controlled trial* or cluster randomi?ed controlled trial* or double blind randomi?ed controlled trial* or randomi?ed consent design or single blind randomi?ed controlled trial* or randomi?ed or placebo or random* or trial or quasi?experiment* or pre*test or post*test or trial or time series or evaluat* or intervention* or "before and after" or intervention* or community trial or non?randomi?ed or repeat* or repeat* measures) or (clinical trial or latin square or random* or clinical trial) or (before NEAR/1 after NEAR/1 (stud* or trial* or design*)) or random* or (quasi?experimental or pseudo?experimental) or (nonrandomi?ed or non?randomi?ed or pseudo?randomi?sed or quasi?randomi?ed) or (population level or population based or population orientated or population oriented or community level or community based or community orientated or community oriented) or (intervention* or prevention or policy or policies or program* or project*)  **Databases:** |
| **S2** | (health promotion) or (health behaviour) or (health behavior) or (policy and (social or school or food or public or urban or environmental or fiscal)) or (urban planning) or (city planning) or (built environment) or (social environment) or (physical environment) or (cultural environment) or (urban environment) or (school environment) or (neighbourhood) or (community) or (societal) or (social interventions) or (community interventions) or (obesogenic environment) or (individual level) or (lifestyle) or (individual) or (tax*) or (subsid*) or (price*) or (health education) or (social marketing) or (diet and (advice or counselling)) or (exercise and (advice or counselling)) or (weight management) or (cash transfer*) or (lifestyle counselling) or (behavioural counselling) or (behavioral counselling) or (exercise on prescription) or (exercise) or (health trainer*) or (school) or (workplace) or (campaign*) or (access NEAR/1 facilities) or (green space) or (walk*ability) or (food label*) or (food advert*)  **Databases:** |
| **S1** | (BMI or Body Mass Index) or (obesity) or (obese) or (overweight) or (weight gain) or (weight loss) or (Body fat) or (Fat mass) or (Weight control*) or (Weight maintain*) or (Adipos*) or (Adipose tissue) or (Skinfold thickness) or (Waist circumference) or (Waist hip ratio) or (WHR)**Databases:** |

**NHS EED (NHS CRD)**

| 1 | descriptor Body Weights and Measures explode all trees in Economic Evaluations |
| --- | --- |
| 2 | MeSH descriptor Obesity explode all trees in Economic Evaluations |
| 3 | BMI or Body Mass Index or Body Weight or obesity or obese or overweight or weight gain or weight loss or Body fat or Fat mass or Weight control* or Weight maintain* or Adipos* or Adipose tissue or Skinfold thickness or Waist circumference. or Waist hip ratio or WHR in Economic Evaluations |
| 4 | (#1 OR #2 OR #3) |
| 5 | MeSH descriptor Health Promotion explode all trees in Economic Evaluations |
| 6 | health promotion or health behaviour or health behavior or (policy and (social or school or food or public or urban or environmental or fiscal)) or urban planning or city planning or built environment or social environment or physical environment or cultural environment or urban environment or school environment or neighbourhood or community or societal or social interventions or community interventions or obesogenic environment or individual level or lifestyle or individual or tax* or subsid* or price* or health education or social marketing or (diet and (advice or counselling)) or (exercise and (advice or counselling)) or weight management or cash transfer* or lifestyle counselling or behavioural counselling or behavioral counselling or exercise on prescription or exercise or health trainer* or school or workplace or campaign* or (access adj1 facilities) or green space or walk?ability or food label* or food advert* in Economic Evaluations |
| 7 | (#5 OR #6) |
| 8 | (evaluat* or effective* or Intervention or RCT or experiment* or randomi?ed controlled trial* or clinical randomi?ed controlled trial* or cluster randomi?ed controlled trial* or double blind randomi?ed controlled trial* or randomi?ed consent design or single blind randomi?ed controlled trial* or randomi?ed or placebo or random* or trial or quasi?experiment* or pre*test or post*test or trial or time series or evaluat* or intervention* or "before and after" or intervention* or community trial or non?randomi?ed or repeat* or repeat* measures) or clinical trial or latin square or random* or exp Evaluation/ or clinical trial or clinical trial.pt. or (before adj1 after adj1 (stud* or trial* or design*)) or random* or (quasi?experimental or pseudo?experimental) or (nonrandomi?ed or non?randomi?ed or pseudo?randomi?sed or quasi?randomi?ed) or ((population level or population based or population orientated or population oriented or community level or community based or community orientated or community oriented) adj3 (intervention* or prevention or policy or policies or program* or project*)) in Economic Evaluations |
| 9 | (#4 AND #7 AND #8) from 2011 to 2012 |
| 10 | (#4 and #7 and #8) from 2011 to 2012 in Economic Evaluations |

**Social Science Citation Index (Web of Science)**

| 1 | TS=(Body Weights and Measures) |
| --- | --- |
| 2 | TS=(BMI or Body Mass Index) or TS=(obesity) or TS=(obese) or TS=(overweight) or TS=(weight gain) or TS=(weight loss) or TS=(Body fat) or TS=(Fat mass) or TS=(Weight control*) or TS=(Weight maintain*) or TS=(Adipos*) or TS=(Adipose tissue) or TS=(Skinfold thickness) or TS=(Waist circumference) or TS=(Waist hip ratio) or TS=(WHR) |
| 3 | #1 or #2 |
| 4 | TS=Health Promotion |
| 5 | TS=(health promotion) or TS=(health behaviour) or TS=(health behavior) or TS=(policy and (social or school or food or public or urban or environmental or fiscal)) or TS=(urban planning) or TS=(city planning) or TS=(built environment) or TS=(social environment) or TS=(physical environment) or TS=(cultural environment) or TS=(urban environment) or TS=(school environment) or TS=(neighbourhood) or TS=(community) or TS=(societal or social interventions) or TS=(community interventions) or TS=(obesogenic environment) or TS=(individual level) or TS=(lifestyle) or TS=(individual) or TS=(tax*) or TS=(subsid*) or TS=(price*) or TS=(health education) or TS=(social marketing) or TS=(diet and (advice or counselling)) or TS=(exercise and (advice or counselling)) or TS=(weight management) or TS=(cash transfer*) or TS=(lifestyle counselling) or TS=(behavioural counselling) or TS=(behavioral counselling) or TS=(exercise on prescription) or TS=(exercise) or TS=(health trainer*) or TS=(school) or TS=(workplace) or TS=(campaign*) or TS=(access N1 facilities) or TS=(green space) or TS=(walk$ability) or TS=(food label*) or TS=(food advert*) |
| 6 | #4 or #5 |
| 7 | TS=(Clinical Trials) OR TS=(Randomized Controlled Trials) or TS=(Double-Blind Studies) or TS=(Single-Blind Studies) or TS=(Crossover Design) |
| 8 | TS=(evaluat* or effective* or Intervention or RCT or experiment* or randomi$ed controlled trial* or clinical randomi$ed controlled trial* or cluster randomi$ed controlled trial* or double blind randomi$ed controlled trial* or randomi$ed consent design or single blind randomi$ed controlled trial* or randomi$ed or placebo or random* or trial or quasi$experiment* or pre*test or post*test or trial or time series or evaluat* or intervention* or "before and after" or intervention* or community trial or non$randomi$ed or repeat* or repeat* measures) or TS=(clinical trial) or TS=(latin square) or TS=(random*) or TS=(clinical trial) or TS=(before N1 after N1 (stud* or trial* or design*)) or TS=(random*) or TS=(quasi$experimental or pseudo$experimental) or TS=(nonrandomi$ed or non$randomi$ed or pseudo$randomi$sed or quasi$randomi$ed) or TS=((population level or population based or population orientated or population oriented or community level or community based or community orientated or community oriented) N3 (intervention* or prevention or policy or policies or program* or project*)) |
| 9 | #7 or #8 |
| 10 | #3 and #6 and #9 |
