# Supplementary material for: A systematic review of the effectiveness of individual, community and societal level interventions at reducing socioeconomic inequalities in obesity amongst children
Source: BMC Public Health. 2014 Aug 11;14:834. doi: 10.1186/1471-2458-14-834 (PMC4137097; doi:10.1186/1471-2458-14-834)
Supplement: Supplementary file 2 — Additional file 2: Effect size tables. Table S1. Intervention effect sizes of the individual level intervention, universal experimental studies. Table S2. Intervention effect sizes of the individual level intervention, targeted (disadvantaged groups only) experimental studies. Table S3. Intervention effect sizes of the community level intervention, universal experimental studies. Table S4. Intervention effect sizes of the community level intervention, targeted (disadvantaged groups only) experimental studies. (DOCX 22 KB) [file 12889_2014_6950_MOESM2_ESM.docx]

**Additional file 2: Effect size tables**

**Table S1: Intervention effect sizes of the individual level intervention, universal experimental studies**

|  | Intervention | | | Control | | |  |  |
| --- | --- | --- | --- | --- | --- | --- | --- | --- |
| Outcome | N | Mean Δ | SD | N | Mean Δ | SD | Effect size | SES analysis |
| *Nutrition and physical activity interventions* | | | | | | | |  |
| **Taveras et al 2011[32]** | | | | | | | | |
| BMI (kg/m^2^) | 160 | 0.27 | 1.39 | 153 | 0.26 | 1.11 | 0.01  (-0.21;0.23) | Higher income group |
| BMI (kg/m^2^) | 88 | 0.4 | 1.59 | 38 | 1.42 | 1.79 | -0.62  (-1.01;-0.23) | Lower income group |
| BMI (kg/m^2^) | 147 | 0.18 | 1.33 | 127 | 0.27 | 1.13 | -0.07  (-0.31;0.17) | Higher education group |
| BMI (kg/m^2^) | 106 | 0.49 | 0.16 | 65 | 0.91 | 1.61 | -0.42  (-0.73;-0.11) | Lower education group |
| **Wake et al 2009[33]** | | | | | | | | |
| BMI (kg/m^2^) | 127 | 0.6 | 2.45 | 115 | 0.7 | 2.16 | -0.04  (-0.29;0.21) | SES did not modify the effect of the intervention on BMI |

**Table S2: Intervention effect sizes of the individual level intervention, targeted (disadvantaged groups only) experimental studies**

|  | Intervention | | | Control | | |  |
| --- | --- | --- | --- | --- | --- | --- | --- |
| Outcome | N | Mean Δ | SD | N | Mean Δ | SD | Effect size |
| *Nutrition and physical activity interventions* | | | | | | | |
| **Black et al 2010[35]** | | | | | | | |
| BMI z score | 89 | 0.01 | 1.19 | 90 | 0.06 | 1.12 | -0.04 (-0.33;0.25) |
| % body fat | 89 | -2.2 | 10.58 | 90 | 1.15 | 11.59 | -0.3 (-0.59;-0.01) |
| Fat mass (kg) | 89 | 0.26 | 11.31 | 90 | 2.71 | 9.1 | -0.24 (-0.53;0.05) |
| FFM (kg) | 89 | 5.68 | 9.95 | 90 | 4.77 | 9.82 | 0.09 (-0.2;0.38) |

**Table S3: Intervention effect sizes of the community level intervention, universal experimental studies**

|  | Intervention | | | Control | | |  |  |
| --- | --- | --- | --- | --- | --- | --- | --- | --- |
| Outcome | N | Mean Δ | SD | N | Mean Δ | SD | Effect size | SES analysis |
| *Nutrition and physical activity interventions* | | | | | | | |  |
| **Kalavainen et al 2007[48]** | | | | | | | | |
| Weight for height | 35 | -6.8 | 6.2 | 35 | -1.8 | 6.2 | -0.8  (-1.29;-0.31) | No association between social class and change in outcomes |
| BMI (kg/m^2^) | 35 | -0.8 | 1 | 35 | 0 | 1.1 | -0.75  (-1.24;-0.26) |  |
| BMI z score | 35 | -0.3 | 0.3 | 35 | -0.2 | 0.3 | -0.33  (-0.8;0.14) |  |

**Table S4: Intervention effect sizes of the community level intervention, targeted (disadvantaged groups only) experimental studies**

|  | Intervention | | | Control | | |  |
| --- | --- | --- | --- | --- | --- | --- | --- |
| Outcome | N | Mean Δ | SD | N | Mean Δ | SD | Effect size |
| *Nutrition only interventions* | | | | | | | |
| **Sichieri et al 2008[45]** | | | | | | | |
| BMI (kg/m^2^) | 434 | 0.32 | 1.49 | 493 | 0.22 | 0.31 | 0.1 (-0.04, 0.24) |
| *Physical activity only interventions* | | | | | | | |
| **Robinson et al 2003[47]** | | | | | | | |
| Waist (cm) | 28 | 0.62 | 14.21 | 33 | 1.08 | 13.26 | -0.03 (-0.54, 0.48) |
| BMI (kg/m^2^) | 134 | 1.28 | 0.9 | 127 | 1.24 | 1.01 | 0.04 (-0.2, 0.28) |
| BMI z score | 134 | 0.26 | 0.19 | 127 | 0.24 | 0.19 | 0.11 (-0.13, 0.35) |
| Waist (cm) | 134 | 4.15 | 2.21 | 127 | 4.25 | 2.54 | -0.04 (-0.28, 0.2) |
| Triceps skinfold (mm) | 134 | 1.49 | 3.01 | 127 | 1.93 | 2.74 | -0.15 (-0.39, 0.09) |
| *Nutrition and physical activity interventions* | | | | | | | |
| **Hamad et al 2011[52]** | | | | | | | |
| BMI z score | 279 | 0.05 | 1.35 | 319 | -0.18 | 1.35 | 0.17 (0.01, 0.33) |
| **Jansen et al 2011 (1)[37]** | | | | | | | |
| BMI (kg/m^2^) | 657 | 0.42 | 2.88 | 729 | 0.5 | 2.93 | -0.03 (-0.13, 0.07) |
| Waist (cm) | 657 | 1.02 | 8.27 | 729 | 2.13 | 8.38 | -0.13 (-0.23, -0.03) |
| **Jansen et al 2011 (2)[37]** | | | | | | | |
| BMI (kg/m^2^) | 583 | 0.76 | 4.11 | 653 | 0.71 | 3.96 | 0.01 (-0.11, 0.13) |
| Waist (cm) | 583 | 2.78 | 11.08 | 653 | 3.43 | 11.14 | -0.06 (-0.18, 0.06) |
| **Kain et al 2004 (3)[36]** | | | | | | | |
| BMI (kg/m^2^) | 1146 | 0 | 3.6 | 491 | 0.3 | 3.2 | -0.09 (-0.19, 0.01) |
| BMI z score | 1146 | -0.12 | 0.95 | 491 | -0.02 | 0.87 | -0.11 (-0.21, -0.01) |
| Triceps skinfold (mm) | 1146 | -0.5 | 6.2 | 491 | -0.8 | 5.75 | 0.05 (-0.05, 0.15) |
| Waist (cm) | 1146 | -0.9 | 10.27 | 491 | 0.9 | 9.1 | -0.18 (-0.28, -0.08) |
| **Kain et al 2004 (4)[36]** | | | | | | | |
| BMI (kg/m^2^) | 995 | 0.3 | 3.85 | 454 | 0.2 | 3.8 | 0.03 (-0.09, 0.15) |
| BMI z score | 995 | -0.04 | 0.9 | 454 | -0.07 | 0.91 | 0.03 (-0.09, 0.15) |
| Triceps skinfold (mm) | 995 | 0.5 | 6.2 | 454 | 0.9 | 6.5 | -0.06 (-0.18, 0.06) |
| Waist (cm) | 995 | 0.8 | 9.9 | 454 | 1.1 | 9.6 | -0.03 (-0.15, 0.09) |
| **Nemet et al 2011[38]** | | | | | | | |
| BMI (kg/m^2^) | 376 | -0.25 | 0.04 | 349 | -0.18 | 0.04 | -1.75 (-1.93, -1.57) |
| BMI % | 376 | -6.4 | 0.8 | 349 | -5.9 | 0.8 | -0.62 (-0.78, -0.46) |
| **Walter et al 1985[46]** | | | | | | | |
| Ponderosity index | 805 | 0.1 | 1.95 | 310 | 0.1 | 1.86 | 0 (-0.14, 0.14) |
| Triceps skinfold (mm) | 805 | 1.1 | 6.25 | 310 | 1.2 | 5.6 | -0.02 (-0.16, 0.12) |
| **Willet 1995[51]** | | | | | | | |
| BMI (kg/m^2^) | 18 | 2.6 | 5.16 | 22 | 2.6 | 7.27 | 0 (-0.63, 0.63) |
| % OW | 18 | 11.9 | 22.91 | 22 | 12.4 | 41.01 | -0.01 (-0.64, 0.62) |
| **De Meij et al 2011[42]** | | | | | | | |
| BMI (kg/m^2^) | 957 | 0.9 | 3.55 | 1099 | 0.7 | 3.55 | 0.06 (-0.02, 0.14) |
| Waist (cm) | 957 | 3.7 | 9.56 | 1099 | 3.3 | 9.2 | 0.04 (-0.04 ; 0.12) |
| **Herrick et al 2012[43]** | | | | | | | |
| BMI (kg/m^2^) | 47 | 0.4 | 0.6 | 51 | 0.2 | 0.7 | 0.31 (-0.08, 0.70) |
| BMI z score | 47 | 0.04 | 0.2 | 51 | 0 | 0.2 | 0.20 (-0.19, 0.59) |
| **Lubans et al 2012[44]** | | | | | | | |
| BMI (kg/m^2^) | 141 | 0.6 | 4.7 | 153 | 0.78 | 4.58 | -0.04 (-0.28, 0.20) |
| BMI z score | 141 | -0.06 | 1.14 | 153 | 0.03 | 1.16 | -0.08 (-0.32, 0.16) |
| Body fat (%) | 141 | 3.14 | 6.2 | 153 | 4.24 | 6.33 | -0.18 (-0.42, 0.06) |

(1) Grades 3-5, (2) Grades 6-8, (3) Boys, (4) Girls
